# Intrafasciomembranal Fluid Pressure: A Novel Approach to the Etiology of Myalgias

**DOI:** 10.7759/cureus.28475

**Published:** 2022-08-27

**Authors:** Stig Runar Hopen

**Affiliations:** 1 Research, HOPEn Muskelterapi, Steinkjer, NOR

**Keywords:** myalgia, myofascial pain syndrome, delayed onset muscle soreness, over training syndrome, chronic exertional compartment syndrome, trigger point, intramuscular fluid pressure, intrafasciomembranal fluid pressure

## Abstract

Fascia is a continuous membrane (fasciomembrane) that enables differentiation of fluid pressure on either side. Fascia membrane also enables an internal increased fluid pressure at all muscle levels (fibers, fiber bundles, skeletal muscles, compartments), and the author introduces a new unifying term for these pressures, regardless of the anatomical level - the intrafasciomembranal fluid pressure (IFMFP). Swelling, pain, and loss of tissue function are identified as common cardinal symptoms in trigger point (TrP), chronic exertional compartment syndrome (CECS), overtraining syndrome (OTS), and delayed onset muscle soreness (DOMS). Existing literature and an overall assessment indicate that intramuscular conditions related to fluid flow and pressure play a central role in different conditions, providing a common biomechanical explanation of the etiology and influence, supporting the article's theory that an increased IFMFP plays a key role in these conditions.

## Introduction and background

Musculoskeletal disorders affect 23% of the general Norwegian population [1]. Lærum et al. in 2013 estimated Norway's socio-economic costs for treatment of musculoskeletal disorders in 2009 were 14.3 billion Norwegian kroner (NOK), of which 2.6 billion accounted for physiotherapists and chiropractors. Further, social security benefits were estimated at NOK 24.6 billion in the form of rehabilitation benefits, time-limited disability benefits, and disability pensions. Thus, the total socio-economic costs were estimated to be between NOK 69 and 73 billion [2]. This is in contrast to a report prepared on behalf of the Norwegian Rheumatism Association (Menon Economics 2019) which estimates the total societal costs for Norway to be over NOK 255 billion in 2016. This report includes loss of income to the state and municipality because of so-called "production loss" as well as the disease burden for the individual - loss of life quality-adjusted years. The Memon report suggests that the social costs are divided between the production loss of NOK 71 billion, total costs for treatment of NOK 18 billion, while the disease burden is estimated at as much as NOK 167 billion [3]. Due to the high costs incurred with soft tissue issues, a clearer understanding of mechanistic disruption is needed.

A part of the musculoskeletal disorders is myalgia, a condition that describes muscle aches and/or pain in soft tissues that often worsen with use and affects approximately 19% of Europeans [4]. Muscle pain typically results from injury associated with repetitive or sustained contraction, e.g., exercise overuse, external trauma, inflammation, ischemia, and in some cases metabolic disorders. Myalgia is often associated with rheumatism, an indefinite term for any chronic or acute condition that is characterized by musculoskeletal inflammation, stiffness, and soreness. Accordingly, myalgia has been referred to as muscular rheumatism [5]. Myalgia is diagnosed by clinical examination, and hypersensitive spots (trigger points) are typical findings [6,7]. These spots are located by palpating within a taut band of muscle fibers [8]. Patients usually present with localized pain in a restricted area or referred pain of various patterns [9]. Interestingly, patients with trapezius fibromyalgia have been found to have higher levels of interstitial lactate and pyruvate [10], and multiple modalities of free nerve ending stimulation (chemical and mechanical) have been implicated with both trauma and inflammation [11]. The literature reviewed for this article suggest that extreme exertion or static muscle loading for extended periods of time may be a cause of myalgia because of impaired fluid flow. Both overtraining, muscle contraction in response to other pains, static work, and stress, all fall under this understanding. Common for these is that the muscle is kept contracted to an excessive degree.

Other conditions that have been likened to myalgias are myofascial trigger point (TrP), chronic exertional compartment syndrome (CECS), overtraining syndrome (OTS), and delayed onset muscle soreness (DOMS). Although both muscular and psychosocial factors have been implicated, the exact etiology of these conditions remains unclear leading to inconsistent diagnostic and treatment strategies. This article aimed to identify similarities in the clinical manifestations between TrP, CECS, OTS, and DOMS. The common denominators can provide a valid experimental model for studying these conditions from a biomechanical and physiological perspective. Cardinal symptoms included swelling, pain, and loss of tissue function, leading to a new approach across diagnoses to provide a common biomechanical explanation of the etiology in the various conditions.

## Review

Fasciomembrane - intrafasciomembranal fluid pressure (IFMFP)

The solid fascia is a continuous membrane that includes endomysium, perimysium, epimysium, and osteo-fascial membrane around muscle compartments [12]. This fascia forms a hierarchy of spaces (small and large compartments) that contain and enclose muscle fibers, fiber bundles, skeletal muscles, and muscle compartments. This membrane enables differentiation of fluid pressure at all levels and enables an increased pressure within such an enclosed space. The author introduces a new unifying term for these pressures, regardless of the anatomical level, the Intrafasciomembranal fluid pressure (IFMFP). Reliable measurements through a cannula have proved difficult as the intramuscular pressure differs in different parts of the muscle [13]. A large number of spaces in a muscle may explain this complexity. Increased fluid pressure is well documented and widely accepted in the CECS and this model is transferable to TrP, OTS, and DOMS. The review of referenced literature in this article as well as the overall assessment supports this new approach.

Trigger point

The myofascial system spreads throughout the body, and the fascia surrounds every muscle and bone, receiving sensory, motor, and autonomic signals alerting the body of pain. Hyperirritable "nodules" known as trigger points (TrP) or myofascial trigger points (mTrP) have been identified and were first described as "stiff, focal 'knots' with exquisite tenderness in a palpable tight skeletal muscle band" [14]. Bron and Dommerholt in 2012 described active and latent mTrP. Active trigger points prevent the muscle from lengthening and weaken the muscle. Latent trigger points are only painful when palpated, and they have a taut band of muscle that increases muscle tension [15]. TrP can occur after acute trauma or by repeated micro-trauma and may develop during occupational, recreational, and physical activity when a muscle is being overused and normal recovery is disturbed. Sustained or repetitive low-level muscle contractions are believed to lead to TrP. TrP has been associated with muscle weakness, fatigue, stiffness, and reduced range of motion [15]. Patients may have one or more TrPs throughout the musculoskeletal system [16]. TrP is also referred as tender point, and some state that these are not synonymous as they define the tender point as a focal node that produced pain directly below the palpation area but don’t cause referred pain [16]. There is a diversity of concepts and some language confusion around the phenomenon TrP/mTRP. The terminology is not very precise, and terms used include muscle infiltration, muscle knot/nodule and in the Scandinavian medical language, the word myose is used. In the broadest sense and to some extent, the term muscle tension is also used [17-19].

Using Doppler ultrasound, Sikdar et al. have quantitatively analyzed blood flow dynamics in blood vessels near TrP to characterize their vascular environment. An increase in the volume of the vascular space and an increased outflow resistance were identified and showed significant differences between active TrP, latent TrP, and normal sites. The flow waveforms near active points showed increased systolic velocities and reversal of flow with negative diastolic velocities [20]. An increased outflow resistance may indicate an accumulation of fluid in the trigger point that inhibits circulation.

Myofascial Pain Syndrome

Myofascial pain syndrome (MPS) is a poorly understood soft-tissue pain syndrome, and TrP is associated with the condition. The prevalence varies from 21% of patients in general orthopedic clinic to as high as 85-93% of patients presenting to specialty pain management centers [20]. MPS is acute, or more commonly, a chronic pain condition. The term MPS is used to describe pain involving the muscle and its surrounding connective tissue e.g. fascia. According to Travell and Simons, TrP is central to the syndrome [21]. However, the diagnostic criteria are subjective and the terminology and models in the scientific literature often disagree [20]. An increased outflow resistance may indicate an accumulation of fluid within the trigger point that inhibits circulation.

Chronic exertional compartment syndrome

Exertional compartment syndrome occurs secondary to elevated intra-compartmental pressure. There are two forms of it, chronic exertional compartment syndrome (CECS) and acute compartment syndrome (ACS). ACS is an emergency that requires acute surgery to avoid limb loss, while CECS is based on a thorough clinical history, with special attention to the patient's characterization of pain during strenuous activity [22]. Increased tissue pressure may be due to a reduction in the size of the fascial membrane or its distensibility (e.g., with a local trauma or inflammation), or be the result of an increase in the volume of the contents. Both can cause the fascia to become tight and restrict the movement of underlying tissue which in turn leads to pain, obstructed range of motion, and decreased blood flow [12,23].

During exercise, the compartment can swell up to 20% secondary to increased blood flow and fluid volume. The increase in pressure in one or more compartments prevents further muscle expansion and the fluid flow is compromised when the volume and pressure reach a level that overrides the capillary perfusion pressure. This increase in pressure will lead to compromised tissue perfusion followed by loss of function, ischemic pain, and neurological symptoms. Subtle motor weakness and/or paresthesia can manifest in the corresponding neurovascular sensory and motor distribution. The pathophysiological cascade due to this abnormal increase in IFMFP results in reduced myocyte oxygenation and ultimately results in myonecrosis and neurological damage [22,24]. McGinley et al. investigated the theory and concluded that CECS occurs as a result of venous outflow obstruction caused by functional muscular compression [25].

Overtraining syndrome

Overtraining is an imbalance between exercise and recovery. Short-term "over-reach" is reversible over days to weeks. Fatigue accompanied by several physical and psychological symptoms in the athlete is an indication of overtraining syndrome (OTS). Dysfunction can occur when physical and emotional stress exceeds the individual's coping capacity. The syndrome and its clinical manifestation can be explained as a stress response [26]. More than 125 signs and symptoms have been identified, making a definitive diagnosis challenging. However, the most common physical symptoms include persistent heavy, stiff and sore muscles, persistent fatigue, washed out/exhausted feeling as well as reduced performance and ability to maintain the training regimen [27].

Ultrasound examination shows significantly greater muscle depth in the vastus lateralis in the overtrained compared to a healthy control group which indicates an increased fluid content in the muscles [28]. Exercise-induced inflammation and cytokine production have been suggested as a possible mechanism behind OTS [29]. The local classic signs of inflammation include pain, swelling, and loss of function, which means that inflammation has many common features with symptoms of exercise-induced muscle damage. Under non-pathophysiological conditions, inflammation is a process in muscle repair and regeneration [29]. With continued intense exercise and the absence of adequate rest, this inflammatory response may be exacerbated, chronic, and pathological [30]. DOMS has been proposed as a possible mechanism underlying skeletal muscle weakness in OTS [30]. Muscle damage, inflammation induced by exercise, and exercise independently cause swelling in the form of increased IFMFP, and this reduced circulation can lead to the water content not being normalized between workouts in OTS.

Delayed onset muscle soreness

Delayed onset muscle soreness (DOMS) is common in people who engage in strenuous and unusual exercise and physical activity [31]. DOMS is a regional pain syndrome and is a common cause of impaired muscle performance in sports and is associated with reduced muscle strength, edema, muscle soreness, and reduced movement. DOMS is considered an ultrastructural muscle injury and the development of exercise-induced intramuscular edema is seen in relation to DOMS [32-34]. The condition causes tenderness and/or stiffness to palpation and/or movement [35]. Excessive physical activity associated with repeated eccentric movements or unusual physical activity in daily living can also cause DOMS.

Several theories are proposed trying to explain the mechanisms underlying DOMS. Lactate accumulation, muscle spasms, muscle damage, connective tissue damage, increased muscle temperature, and inflammation are included theories [31]. The inflammation theory suggests that DOMS reflects events commonly seen in acute inflammation, including cardinal symptoms of pain, swelling, and loss of function [34]. Swelling, intramuscular edema, and inflammation are commonly mentioned in literature dealing with DOMS, and this coincides with increased IFMFP.

Intrafasciomembranal fluid pressure as a unifying model

Muscle use that exceeds the muscle's capacity and disturbed recovery (mechanical muscle overuse, static or repetitive) seem to be accepted as mechanisms in TrP, CECS, OTS, and DOMS [15,22,26,31]. These conditions have overlapping symptoms and may be seen as expressions of the common mechanism of IFMFP. The symptoms are often described as persistent swelling/intramuscular edema, pain, stiffness/soreness to palpation and/or movement, motor weakness, and obstructed range of motion. They often worsen with stimuli such as increased activity and/or palpation [12,16,20,22-24,27,30,32-35].

Fluid in each muscle fiber can be a source of muscle tension and myalgias and play a key role in the mechanics behind these conditions. Pressure can have immediate, significant effects on muscle contractile force and suggests that forces transmitted to the extracellular matrix via fluid under pressure may be important, but largely unknown [36-38]. An increased fluid volume within a fascial membrane, regardless of anatomical level, thus results in an increased IFMFP that mechanically may restrict mobility, making the tissue "inflated" and stiff. These pressures are rarely considered in mechanical models of muscles but have the potential to affect performance by affecting the force and work produced during contraction [38].

Muscle fluid shifts

It is likely that all forms of muscle use affect the volume of fluid in the muscles. Water is distributed in the extracellular and intracellular space and the exchanges are mainly controlled by osmotic pressure and it determines the volume of the cell [39]. Capillary blood flow is temporarily blocked during muscle contractions and resumes with relaxation, in a mechanism known as the muscle pump [15]. Muscle activity requires adjustments in the cardiovascular system to meet the needs for increased blood flow in the contracting skeletal muscles. Exercise hyperemia refers to the increase in blood flow in the skeletal muscles that occurs during muscle activity. Arterial inflow to active muscles decreases during contractions and increases when skeletal muscle relaxes. On the other hand, venous outflow increases during rhythmic contractions, but decreases during muscle relaxation. These mechanical effects of muscle activity are due to increased extravascular pressure during rhythmic contractions [40].

Compromised circulation

The water content of the skeletal muscles is, under normal conditions, reduced and normalized after the activity ceases. Overuse or intense use over time may impair venous outflow and fluid volume remains elevated because of insufficient amounts of fluid drainage between contractions. Even relatively small voluntary contractions can cause intramuscular pressure high enough to significantly impair intramuscular blood circulation [15].

Swelling, pain, and loss of tissue function

The overlapping symptoms of swelling, pain, and loss of tissue function seen in TrP, CECS, OTS, and DOMS have common features with inflammation. Local inflammatory and immune responses alter vascular permeability [41], which normally regulates interstitial pressure in the tissue [42]. Inflammation is a recognized key process in muscle repair and regeneration and ultimately leads to adaptive rebuilding. This mitigating response contributes to the restoration of tissue homeostasis and resolution of the inflammation [29]. Acute and chronic, systemic, and focal inflammation can be low-grade as observed in myalgia. The symptoms coincide with those of TrP, CECS, OTS, and DOMS, and we can assume that even a moderate associated swelling may impair local circulation, additionally predisposing to ischemic pain and mechanical stimuli of pain receptors [41,43].

Increased blood flow and fluid volume in CECS is what ultimately results in an increase in pressure, and this prevents further muscle expansion. The IFMFP compromises blood flow when the volume and pressure reach a level that overrides capillary perfusion pressure, followed by motor weakness and/or paresthesia, loss of function, ischemic pain, and neurological symptoms [22,24]. CECS may be secondary to increased fluid content, which impairs muscle/nerve function, or to nerves not receiving adequate perfusion secondary to elevated pressure that ultimately compromises small capillary flow [23,25]. Increased IFMFP is in any case related to the pathology of the CECF and provides a functional muscular compression which in turn leads to venous outflow obstruction [24]. Such outflow obstruction has also been observed using Doppler ultrasound in blood vessels near the TrP. An increase in the volume of the vascular space leads to a narrowed vascular system and thus gives an increased outflow resistance [20]. It is conceivable that the lymph capillaries also partially collapse under this IFMFP, and fluid and waste products will accumulate in the tissue since drainage of the tissue fluid is distributed between venous blood and lymph capillaries, leading to compromise backflow and escalating circulatory dysfunction [44]. Consequences on immune cell transport can be especially harmful during inflammation and compromise edema resolution [45].

Sikdar et al. suggest that the increased outflow resistance may be due to muscle contractions in TrP that compress the capillary or venous bed [20]. When the contraction in the muscle ceases, the increased IFMFP itself may be of such a nature that it compresses the capillary or venous bed in TrP, CECS, OTS, and DOMS. The fascia may also lose its flexibility following inflammation (swelling), restricting the movement of underlying tissue [12]. Free movement is necessary to maintain normal metabolism. Elevated IFMFP may also cause loss of tissue function by reducing the muscle's mechanical ability to contract, similarly to the reduction of the muscle's total contractile ability through swelling, prior to voluntary contraction. Whatever the cause, the result is muscle weakness, reduced movement, and compromised circulation due to swelling, pain, and loss of tissue function.

## Conclusions

This article aimed to identify similarities in the clinical manifestations between TrP, CECS, OTS, and DOMS. Compartment is used as a unifying model for the mechanisms of all these condition. CECS is due to increased intramuscular pressure that causes swelling which impairs the circulation, cause ischemic pain and affect the metabolism. Muscle fibers, fiber bundles, skeletal muscles, and compartments are surrounded by their own fascia membrane and may all potentially be exposed to increased intrafasciomembranal fluid pressure (IFMFP). Given that free nerve endings (nociceptors) terminate in the interstitial space close to the muscle fibers, any changes in fluid volume could stimulate pain. Increased IFMFP can occur in part of or the entire skeletal muscle as well as in the compartments. As the symptoms (swelling, pain, and loss of tissue function) of TrP, CECS, OTS, and DOMS coincide, and DOMS is seen in connection with both TrP and OTS, and since venous outflow obstruction is observed in both TrP and CECS, this review indicates that an increased IFMFP play a key role in TrP, CECS, OTS, and DOMS. The common denominators derived from IFMFP leading to an approximation across diagnoses to provide a common biomechanical explanation of the etiology in the various conditions and provides a possible explanation for the "knots" and "threads," the edematous and hypertonic muscles reported. Further research is recommended.

## References

[1] Kinge JM, Knudsen AK, Skirbekk V, Vollset SE (2015). Musculoskeletal disorders in Norway: prevalence of chronicity and use of primary and specialist health care services. BMC Musculoskelet Disord.

[2] Lærum E (2014). A Musculoskeletal Account: Incidence and Costs Related to Injuries, Diseases and Ailments in the Musculoskeletal System. Muskel- og skjelett tiåret v/FORMI, Klinikk for kirurgi og nevrofag, Oslo universitetssykehus, Ullevål. 
Page 16.

[3] (2022). Social costs related to muscle and bone disease. https://www.menon.no/publication/samfunnskostnader-knyttet-muskel-skjelettsykdom/.

[4] Breivik H, Collett B, Ventafridda V, Cohen R, Gallacher D (2006). Survey of chronic pain in Europe: prevalence, impact on daily life, and treatment. Eur J Pain.

[5] Bockman CS, Eckerson J, McCarson KE (2015). Myalgia. Reference Module in Biomedical Sciences.

[6] Nesbit SP (2007). The disease control phase of treatment. Treatment Planning in Dentistry. Second Edition.

[7] (2022). Myalgia. https://www.sciencedirect.com/topics/neuroscience/myalgia.

[8] Dommerholt J, Bron C, Franssen J (2006). Myofascial trigger points: an evidence-informed review. J Man Manip Ther.

[9] Tantanatip A, Chang Ke-Vin (2022). Myofascial pain syndrome. StatPearls [Internet].

[10] Gerdle B, Söderberg K, Puigvert LS, Rosendal L, Larsson B (2010). Increased interstitial concentrations of pyruvate and lactate in the trapezius muscle of patients with fibromyalgia: a microdialysis study. J Rehabil Med.

[11] Mense S (2009). Algesic agents exciting muscle nociceptors. Exp Brain Res.

[12] Gatt A, Agarwal S, Zito PM (2022). Anatomy, fascia layers. StatPearls [Internet].

[13] Nakhostine M, Styf JR, van Leuven S, Hargens AR, Gershuni DH (1993). Intramuscular pressure varies with depth. The tibialis anterior muscle studied in 12 volunteers. Acta Orthop Scand.

[14] Travell J, Rinzler S, Herman M (1942). Pain and disability of the shoulder and arm: treatment by intramuscular infiltration with procaine hydrochloride. J Am Med Assoc.

[15] Bron C, Dommerholt JD (2012). Etiology of myofascial trigger points. Curr Pain Headache Rep.

[16] Hammi C, Schroeder JD, Yeung B (2022). Trigger point injection. StatPearls [Internet].

[17] Kåss E (2019). Miosis. http://sml.snl.no/myoser.

[18] Lings S (2005). 2004 myoses. [Article in Danish]. Ugeskr Laeger.

[19] Hansen SE (2005). The Danish myosis 2004. [Article in Danish]. Ugeskr Laeger.

[20] Sikdar S, Ortiz R, Gebreab T, Gerber LH, Shah JP (2010). Understanding the vascular environment of myofascial trigger points using ultrasonic imaging and computational modeling. Annu Int Conf IEEE Eng Med Biol Soc.

[21] Shah JP, Thaker N, Heimur J, Aredo JV, Sikdar S, Gerber L (2015). Myofascial trigger points then and now: a historical and scientific perspective. PM R.

[22] Chandwani D, Varacallo M (2022). Exertional compartment syndrome. StatPearls [Internet].

[23] Braver RT (2016). Chronic exertional compartment syndrome. Clin Podiatr Med Surg.

[24] Liu B, Barrazueta G, Ruchelsman DE (2017). Chronic exertional compartment syndrome in athletes. J Hand Surg Am.

[25] McGinley JC, Thompson TA, Ficken S, White J (2022). Chronic exertional compartment syndrome caused by functional venous outflow obstruction. Clin J Sport Med.

[26] Kuipers H, Keizer HA (1988). Overtraining in elite athletes. Review and directions for the future. Sports Med.

[27] Roy BA (2015). Overreaching/overtraining: more is not always better. ACSMs Health Fit J.

[28] Høgseth J, Omfjord CS, Dahl HA (2005). Feiltrening hos idrettsutøvere: drøfting av en fysioterapitilnærming. Fysioterapeuten.

[29] Cheng AJ, Jude B, Lanner JT (2020). Intramuscular mechanisms of overtraining. Redox Biol.

[30] Kreher JB, Schwartz JB (2012). Overtraining syndrome: a practical guide. Sports Health.

[31] Ranchordas MK, Rogerson D, Soltani H, Costello JT (2017). Antioxidants for preventing and reducing muscle soreness after exercise. Cochrane Database Syst Rev.

[32] Vadasz B, Gohari J, West DW (2020). Improving characterization and diagnosis quality of myofascial pain syndrome: a systematic review of the clinical and biomarker overlap with delayed onset muscle soreness. Eur J Phys Rehabil Med.

[33] Heiss R, Hotfiel T, Kellermann M (2018). Effect of compression garments on the development of edema and soreness in delayed-onset muscle soreness (DOMS). J Sports Sci Med.

[34] Smith LL (1991). Acute inflammation: the underlying mechanism in delayed onset muscle soreness?. Med Sci Sports Exerc.

[35] Cheung K, Hume P, Maxwell L (2003). Delayed onset muscle soreness: treatment strategies and performance factors. Sports Med.

[36] Joyner MJ, Casey DP (2015). Regulation of increased blood flow (hyperemia) to muscles during exercise: a hierarchy of competing physiological needs. Physiol Rev.

[37] Sleboda DA, Roberts TJ (2017). Incompressible fluid plays a mechanical role in the development of passive muscle tension. Biol Lett.

[38] Sleboda DA, Roberts TJ (2020). Internal fluid pressure influences muscle contractile force. Proc Natl Acad Sci U S A.

[39] Lorenzo I, Serra-Prat M, Yébenes JC (2019). The role of water homeostasis in muscle function and frailty: a review. Nutrients.

[40] Korthuis RJ (2011). Exercise hyperemia and regulation of tissue oxygenation during muscular activity. Skeletal Muscle Circulation.

[41] Chen L, Deng H, Cui H (2018). Inflammatory responses and inflammation-associated diseases in organs. Oncotarget.

[42] Claesson-Welsh L (2015). Vascular permeability--the essentials. Ups J Med Sci.

[43] Pahwa R, Goyal A, Jialal I (2022). Chronic inflammation. StatPearls [Internet].

[44] Moore JE Jr, Bertram CD (2018). Lymphatic system flows. Annu Rev Fluid Mech.

[45] von der Weid PY, Rehal S, Dyrda P (2012). Mechanisms of VIP-induced inhibition of the lymphatic vessel pump. J Physiol.

